# The Role of Leptin Levels in Adaptation to Cold Climates

**DOI:** 10.3390/ijerph17061854

**Published:** 2020-03-12

**Authors:** Alena A. Nikanorova, Nikolay A. Barashkov, Sergey S. Nakhodkin, Vera G. Pshennikova, Aisen V. Solovyev, Georgii P. Romanov, Sargylana S. Kuzmina, Nikolay N. Sazonov, Tatyana E. Burtseva, Jon Øyvind Odland, Sardana A. Fedorova

**Affiliations:** 1 Laboratory of Molecular Genetics, Yakut Science Centre of Complex Medical Problems, 677010 Yakutsk, Sakha Republic (Yakutia), Russia; nikanorova.alena@mail.ru (A.A.N.); sergnahod@mail.ru (S.S.N.); psennikovavera@mail.ru (V.G.P.); nelloann@mail.ru (A.V.S.); gpromanov@gmail.com (G.P.R.); sardaanafedorova@mail.ru (S.A.F.); 2 Laboratory of Molecular Biology, M.K. Ammosov North-Eastern Federal University, Yakutsk, 677000 Sakha Republic (Yakutia), Russia; sskuzmina@bk.ru (S.S.K.); saznilol@mail.ru (N.N.S.); 3 Laboratory of the Human in the Arctic, The Institute for Humanities Research and Indigenous Studies of the North, Federal Research Center “Yakut Science Center of Siberian Branch of Russian Academy of Science”, Yakutsk, 677027 Sakha Republic (Yakutia), Russia; 4 Department of Pediatrics and Child Surgery, M.K. Ammosov North-Eastern Federal University, Yakutsk, 677000 Sakha Republic (Yakutia), Russia; bourtseva@yandex.ru; 5 Laboratory of Monitoring Children Health and Medico-environmental Research, Yakut Science Centre of Complex Medical Problems, Yakutsk, 677010 Sakha Republic (Yakutia), Russia; 6 Department of Public Health and Nursing, Faculty of Medicine and Health Sciences, NTNU Norwegian University of Science and Technology, 7003 Trondheim, Norway; jon.oyvind.odland@uit.no

**Keywords:** leptin, meta-analysis, thermoregulation, cold climates, adaptation

## Abstract

Currently, adipose tissue is considered an endocrine organ that produces hormone-active substances, including leptin, which can play a key role in thermoregulation processes. Therefore, we performed a meta-analysis to investigate the influence of the climatic environment on leptin levels. A systematic literature search in the databases was carried out on 10 January 2020. Finally, 22 eligible articles were included in the current meta-analysis and a total of 13,320 participants were covered in the final analysis. It was shown that males of the “North” subgroup demonstrated significantly higher levels of leptin (10.02 ng/mL; CI: 7.92–12.13) than males of the “South” subgroup (4.9 ng/mL; CI: 3.71–6.25) (*p* = 0.0001). On the contrary, in the female group, a similar pattern was not detected (*p* = 0.91). Apparently, in order to maintain body temperature, higher leptin levels are required. The results of the study indicate that such effects are most pronounced in males and to a smaller extent in females, apparently due to a relatively high initial concentration of leptin in females. The correlation between leptin levels and climatic environment data support the hypothesis of leptin-mediated thermoregulation as an adaptive mechanism to cold climates.

## 1. Introduction

The indigenous people living in circumpolar regions have certain anatomical and physiological adaptations to protect the body from prolonged exposure to cold [1,2,3]. In response to chronic cold exposure, the rate of energy expenditure must increase to generate the additional heat needed to avert a drop in body temperature. One of the main mechanisms of increasing heat production in the body is nonshivering thermogenesis. This is mainly due to the metabolism in brown adipose tissue (BAT). Although BAT was initially considered to be present only in infants, it is now established that substantial BAT depots can be detected in the supraspinal, supraclavicular, pericardial and neck regions of adult humans [4,5,6,7,8,9]. In 2015, BAT in adults living in the cold climatic conditions of Eastern Siberia (Sakha Republic, Russia) was found. The BAT was detected in samples of adipose tissue from paraaortic, perirenal, subclavian, and parathyroid areas [10]. In BAT, nonshivering thermogenesis is mediated primarily by the uncoupling protein 1 (UCP1). This protein uncouples fatty acid oxidation from adenosine triphosphate production, leading to a futile metabolic process that results in increased heat production [11]. According to Efremova et al. [12], it was found that a constant part of the mediastinal and perirenal fat (up to about 40%) in adult residents of Eastern Siberia had a morphology typical of brown adipocytes and that a relevant percentage of it (up to about 30%) expressed the functional marker of UCP1 [12]. Many studies have shown that prolonged exposure to cold temperatures results in white-to-brown adipocyte transdifferentiation (browning) [13,14,15,16]. The process of browning is currently being studied by many researchers around the world [15,17,18,19,20,21,22,23,24,25,26,27,28]. One of the most relevant in this sphere is the study of hormonal activities in browning processes. Leptin was the first adipokine to be discovered [28]. In humans, leptin is the product of the *LEP* gene that is located on chromosome 7 [29]. Leptin plays an important role in regulating energy homeostasis, but it can also affect some other physiological processes [30,31,32,33]. It was found that leptin can increase the expression of UCP1 [34,35], and stimulate the oxidation of fatty acids [36,37]. Leptin-deficient ob/ob mice are characterized not only by hyperphagia and obesity but also mild hypothermia; such mice will not survive a prolonged cold exposure [38]. Subsequently, it was found that the administration of exogenous leptin can optimize the body temperature in ob/ob mice [39], which suggests the direct involvement of leptin in thermoregulation. The available data suggest that leptin is involved in thermoregulation and possibly in adaptation to cold climates. Therefore, the present systematic review and meta-analysis were performed to summarize the results of original articles into a quantitative estimation of the correlation between leptin levels and climatic environment.

## 2. Materials and Methods

The present study was conducted following the Preferred Reporting Items of Systematic Reviews and Meta-Analyses statement [40].

### 2.1. Search Strategy

PubMed-Medline databases were searched by using the following search terms in titles and abstracts: (“leptin”[MeSH Terms] OR “leptin”[All Fields]) AND (“body mass index”[MeSH Terms] OR (“body”[All Fields] AND “mass”[All Fields] AND “index”[All Fields]) OR “body mass index”[All Fields] OR (“body”[All Fields] AND “mass”[All Fields] AND “index”[All Fields] AND “bmi”[All Fields]) OR “body mass index bmi”[All Fields]). The literature was searched from inception to 10 January 2020.

### 2.2. Inclusion and Exclusion Criteria

The inclusion criteria for the studies were as follows: (1) the studies were case-controlled, cross-sectional, prospective, or clinical trials; (2) the studies examined serum or plasma leptin levels; (3) blood leptin levels should be measured using an enzyme-linked immunosorbent assay (ELISA) or radioimmunoassay (RIA), as these two methods are highly sensitive and specific; (4) leptin level unit is ng/mL; (5) the studies used mean values and standard deviations with a confidence interval (95%). 

The exclusion criteria of studies were: (1) uncontrolled trials; (2) lack of sufficient information on baseline or follow-up leptin concentrations; (3) duplicated study.

### 2.3. Data Extraction

Eligible studies were reviewed, and the following data were abstracted: (1) author’s name; (2) year of publication; (3) country where the study was performed; (4) group size; (5) mean age or age range; (6) gender; (7) body mass index; (8) leptin levels; (9) blood leptin levels measurement method.

### 2.4. Quality Control

The methodological quality of the included studies was evaluated by the Newcastle-Ottawa scale. A total of nine items were involved in this form. The high-quality study was defined as a study with ≥7 awarded stars.

### 2.5. Data Analysis

Meta-analysis was conducted using RevMan 5.3 (The Cochrane Collaboration). The difference in blood leptin levels in individuals with elevated leptin and control groups was estimated by calculating the mean difference (MD). If the χ^2^ value was less than 0.10 and I^2^ exceeded 50%, then we considered there to be substantial heterogeneity and a random-effect model was applied to pool the data.

### 2.6. Climatic Environment

**Figure 1:**
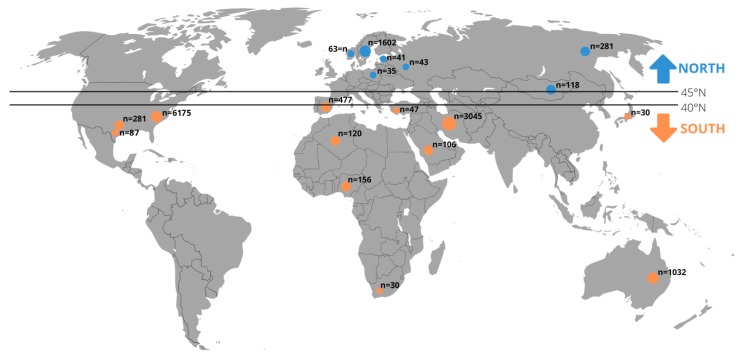
The 13,320 individuals divided into the “North” and “South” subgroups included in the meta-analysis (detailed description about the literature search and eligible studies can be found in the results in Section 3.1).

We divided data of included original articles by the climatic environment into “North” and “South” subgroups. The division into “North” and “South” was made according to the approximate border (40–45° N) of the transition from the subtropical climate zone to the temperate climate zone (Figure 1).

### 2.7. Limitations of Meta-Analysis

There are certain limitations to our study. First, some heterogeneity may lead to reduced statistical power. Second, because sufficient data in primary studies are lacking, we were unable to perform further analyses to investigate other factors, such as a body fat mass, age, smoking, physical activity, diet, which may have affected our results.

### 2.8. Ethics Statement

This study was approved by the local Biomedical Ethics Committee of Yakut Scientific Center of Complex Medical Problems, Siberian Branch of the Russian Academy of Medical Sciences, Yakutsk, Russia (Yakutsk, Protocol No. 16, 13 December 2014).

## 3. Results

### 3.1. Literature search and Eligible Studies

**Figure 2:**
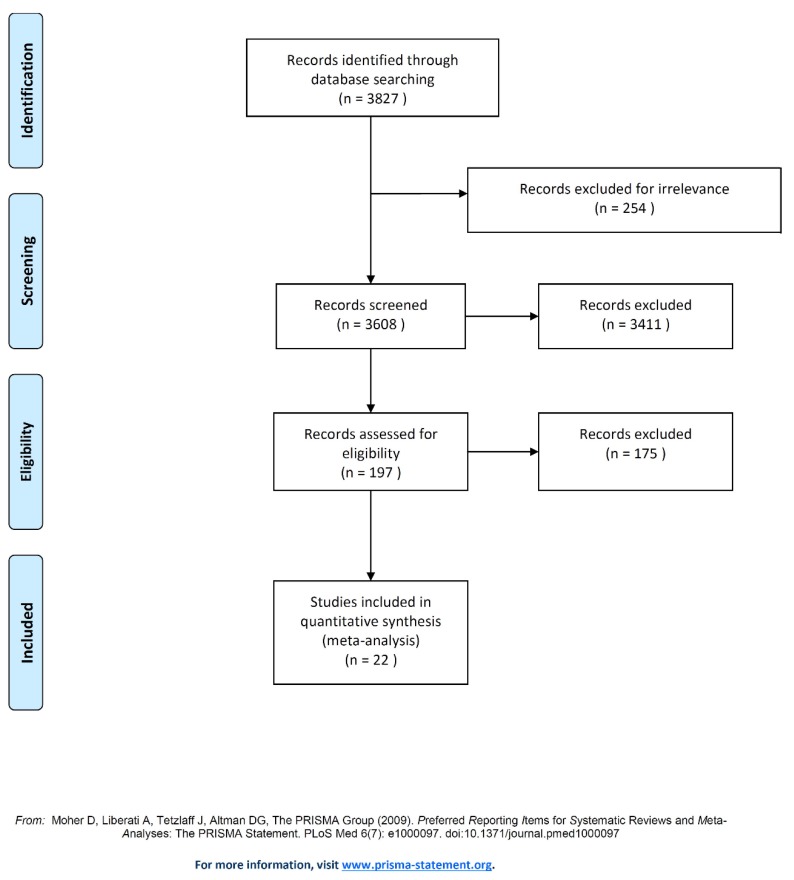
Flow chart of study selection. Note: Presentation of the process by which relevant studies were retrieved from the databases, assessed, and selected, or excluded. Preferred reporting items for systematic reviews and meta-analyses (PRISMA) diagram for the study search [40].

The search of the literature yielded 3827 relevant articles from PubMed. Then, the abstracts of these articles were reviewed to assess their eligibility. Following this assessment, 3608 articles did not meet the inclusion criteria, hence they were excluded. Furthermore, full texts of the 219 articles were reviewed and this resulted in the exclusion of 197 articles. In the end, 22 eligible articles met the inclusion criteria and were included in the final meta-analysis (Figure 2) [41,42,43,44,45,46,47,48,49,50,51,52,53,54,55,56,57,58,59,60,61,62].

### 3.2. Characteristics of the Articles

The final analysis included a total of 13,320 participants with sample sizes ranging from 15 to 1507 in the individual studies. These studies were published between 1994 and 2020. The age of the participants ranged from 20 to over 80 years. The detailed characteristics of these studies are summarized in Table 1. The control group included 9390 individuals (3840 females, 5550 males) with normal body weight (18.5–24.99 kg/m^2^). The group with elevated leptin levels included 3930 individuals (1933 females, 1997 males) with excess body weight and obesity (25–35 kg/m^2^). The data were divided into two subgroups (“North” and “South”), according to the geographical zones of the research sites.

### 3.3. The Leptin Levels Depending on Climatic Conditions

**Figure 3:**
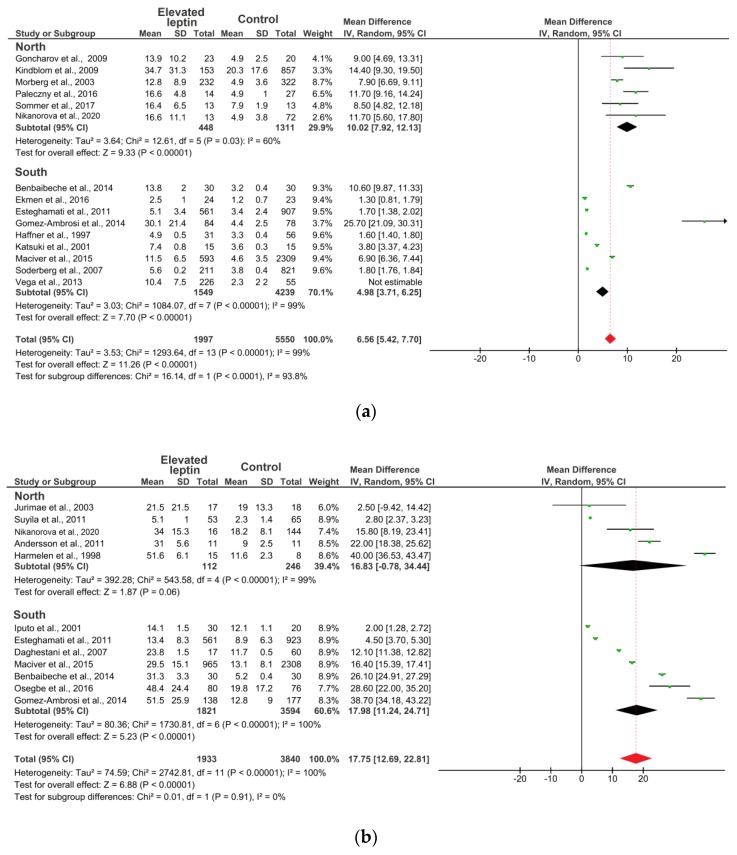
Forest plot of meta-analysis: Mean difference in the circulating level of leptin in subjects with elevated leptin and controls: (**a**) males; (**b**) females. **Note:** Mean—average value; SD—standard deviation; Dotted line—the point estimate of the averaged studies; Black diamond—the point estimation; Red diamond—the average point estimation.

Figure 3 presents a forest plot for the MD constructed from a random-effects model of the leptin levels between elevated leptin subjects and controls in the 22 eligible studies. In Figure 3a, in males’ subgroup, the point estimate (black diamond’s) does not intersect with the point estimate of the averaged studies (dotted line), which indicates that there are statistically significant differences. For females, the point estimate (black diamond’s) of the “North” and “South” groups intersect with the point estimate of the averaged studies (dotted line), which indicates that there are no significant differences (Figure 3b). Thus, analysis of subgroups by leptin levels depending on the climatic environment revealed statistically significant differences among the males of the “North” subgroup, where the average leptin level (10.02 ng/mL; CI: 7.92–12.13) was twice as high compared to the “South” subgroup (4.98 ng/mL; CI: 3.71–6.25) (*p* = 0.0001) (Figure 3a). However, no statistically significant differences were found in the females (*p* = 0.91) (Figure 3b).

## 4. Discussion

**Figure 4:**
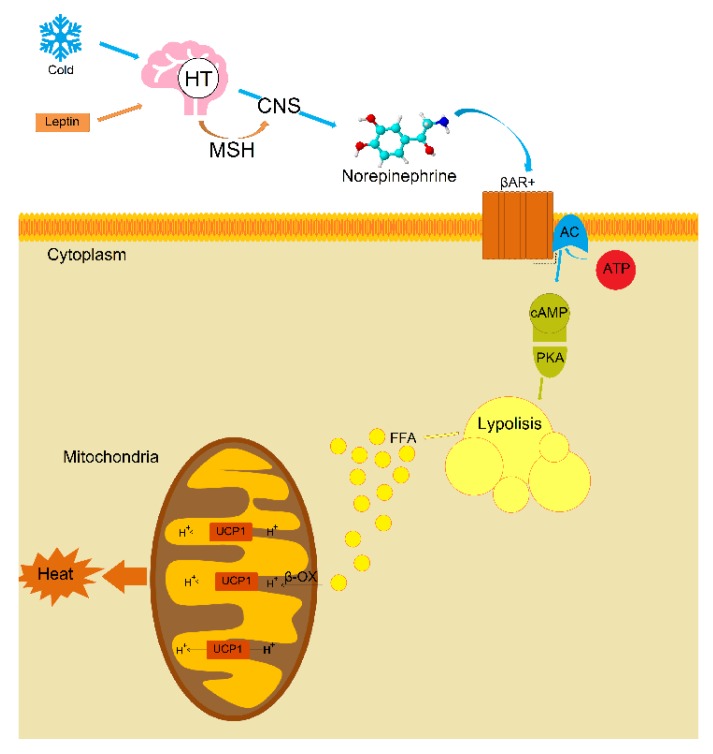
Possible mechanism of leptin-dependent neuro-fatty linkage of nonshivering thermogenesis in brown adipose tissue (The figure adapted from the articles: [64,65,67]). **Note:** HP—hypothalamus, MSH—α-melanocyte-stimulating hormone, CNS—sympathetic nervous system, βAR—β-adrenaline receptors, AC—adenylate cyclase, ATP—adenosine triphosphate, cAMP—cyclic adenosine monophosphate, PKA—protein kinase A, FFA—free fatty acids, β-OX—β-oxidation.

For the first time, using published data, the meta-analysis was carried out to assess the influence of climatic conditions on the levels of leptin circulating in the blood of 13,320 individuals. The obtained results showed significant differences in leptin levels between “northern” and “southern” males, indicating the involvement of leptin in the key processes of thermoregulation and possibly in the mechanisms of human adaptation to cold environment. It is known that the mechanism of the regulation of thermoregulation and browning by leptin is associated with neurons of pro-opiomelanocortin (POMC) in the arcuate nucleus [50,51,52,53]. Through the mechanism of leptin-dependent neuro-adipose linkage of nonshivering thermogenesis in BAT (Figure 4), leptin and its receptors in the arcuate nucleus of the hypothalamus increase the activity of POMC neurons, triggering the production and release of α-melanocyte-stimulating hormone. This hormone activates melanocortin-3 and 4 receptors, which increases the activity of the sympathetic nervous system and leads to an increased expression of uncoupling protein UCP1 in BAT [31,63]. In the BAT, noradrenaline is released as a response to signals from the hypothalamus which is activated by cold receptors in the skin. Furthermore, in the outer membrane of the brown fat cells, noradrenaline activates—via β-adrenalgenic receptors—adenylate cyclase in the cytosol of these cells to form cAMP. Via a protein kinase cascade, cAMP activates triglyceride lipase so that free fatty acids are formed. The released free fatty acids react with UCP1 and overcome UCP1 inhibition, wherein all the energy from the combustion of the substrate (food) is directly released as heat [64,65,66]. 

The involvement of leptin in thermoregulation and energy homeostasis was also previously shown [38,39,68,69]. A study by Farooqi et al. [70] found that people with congenital leptin deficiency have mild hypothermia and low energy expenditure [70]. The body temperature and energy expenditure in these individuals were normalized with the introduction of exogenous leptin [70]. It can be assumed that, under the influence of low temperatures on the body, relatively high levels of leptin in the blood prevent a decrease in energy expenditure and body temperature, which, in turn, can be an adaptogenic mechanism to the effects of cold. The results of our study indicate that such effects are most pronounced in males and to a smaller extent in females, since it is known that females have a relatively high initial concentration of this adipokine in the blood due to their physiological features [70]. It was also previously suggested that women have relatively high levels of leptin due to a lower sensitivity to leptin in the central nervous system [71], as was shown in most obese humans having a leptin resistance [72]. The obtained results of the impact of the climatic environment on leptin levels suggest a possible leptin-dependent neuro-adipose connection between nonshivering thermogenesis and browning in people living in a cold climatic environment.

## 5. Conclusions

In this study, we showed a correlation between leptin levels and climatic environment data supporting the hypothesis of leptin-mediated thermoregulation as an adaptive mechanism to cold climates.

## Figures and Tables

**Table 1:** Characteristics of the individual studies included in this meta-analysis.

Author Publication, Year	Country/Setting	BMI (Normal), kg/m^2^	Leptin Level, ng/mL	Subjects (n)/Age	BMI (Overweight/Obese), kg/m^2^	Leptin Level, ng/mL	Subjects (n)/Age	ELISA or RIA	Serum or Plasma	Subgroups
FEMALES										
Nikanorova et al., 2020	Russia	21.42 ± 1.33	18.18 ± 8.13	144/19.81 ± 2.09	27.56 ± 2.21	34 ± 15.3	16/20.56 ± 2.4	ELISA	Serum	North
Suyila et al., 2011	Mongolia	21.75 ± 1.9	2.35 ± 1.39	65/31.67 ± 3.87	30 ± 3.3	5.09 ± 1.07	53/41.54 ± 3.78	ELISA	ND	North
Andersson et al., 2011	Sweden	21 ± 0.6	9 ± 2.5	11/ND	31 ± 2.1	31 ± 5.6	11/ND	RIA	ND	North
Harmelen et al., 1998	Sweden	22.3 ± 0.9	11.6 ± 2.3	8/45 ± 3	41.9 ± 2.3	51.6 ± 6.1	15/43 ± 3	RIA	Serum	North
Jurimae et al., 2003	Estonia	23.8 ± 1.7	19 ± 13.3	18/67.1 ± 6.4	31.4 ± 4.7	21.5 ± 21.5	17/67.0 ± 6.9	RIA	Plasma	North
Iputo et al., 2001	RSA	23 ± 4.5	12.1 ± 1.1	20/20	29.8 ± 5	14 ± 1.2	10/35	ELISA	Serum	South
Daghestani et al., 2007	Saudi Arabia	20.85 ± 0.25	11.7 ± 0.46	60/23.95 ± 0.6	35.9 ± 0.92	46.04 ± 3.07	46/26.49 ± 0.96	ELISA	Serum	South
Osegbe et al., 2016	Nigeria	21 ± 0.6	19.8 ± 17.2	76/ND	39.1 ± 7.2	48.4 ± 24.4	80/44.9 ± 9.8	ELISA	Serum	South
Gomez-Ambrosi et al., 2014	Spain	22.7 ± 1.7	12.8 ± 9	177/47.0 ± 10.0	39.7 ± 7	51.5 ± 25.9	138/47.2 ± 10.2	RIA	ND	South
Maciver et al., 2015	USA	24.3 ± 3.3	13.1 ± 8.1	2308/46.4 ± 18.9	35.5 ± 5	29.5 ± 15.1	965/46.5 ± 16.8	RIA	Serum	South
Benbaibeche et al., 2014	Algeria	20 ± 2	5.22 ± 0.4	30/35 ± 4	34 ± 1	31.35 ± 3.3	30/40 ± 3	ELISA	ND	South
Esteghamati et al., 2011	Iran	25.46 ± 4.89	8.99 ± 6.34	923/40.21 ± 11.42	31.24 ± 5.21	13.38 ± 8.31	654/48.23 ± 10.28	ELISA	Serum	South
MALES										
Sommer et al., 2017	Norway	23.6 ± 2	7.9 ± 1.9	50/50 + 7	29.5 ± 2.3	16.4 ± 6.5	13/53 + 6	ELISA	Plasma	North
Paleczny et al., 2016	Poland	25.1 ± 2.7	4.9 ± 1.02	27/46 ± 8	33.7 ± 3.5	16.6 ± 4.83	14/44 ± 8	ELISA	Plasma	North
Nikanorova et al., 2020	Russia	21.96 ± 1.68	7.51 ± 1.13	72/20 ± 1.67	26.64 ± 1.17	8.48 ± 1.61	13/20 ± 1.21	ELISA	Serum	North
Kindblom et al., 2009	Sweden	25.9 ± 3.3	20.3 ± 17.6	857/75.3 ± 3.2	27.7 ± 4.2	34.7 ± 31.3	153/75.1 ± 3.3	ELISA	Serum	North
Morberg et al., 2003	Sweden	26.1 ± 3.7	4.9 ± 3.6	323/49.9 ± 6.0	35.9 ± 5.9	12.8 ± 8.9	234/47.5 ± 5.1	RIA	Serum	North
Goncharov et al., 2009	Russia	23.1 ± 1.3	4.9 ± 2.5	20/20–40	30.8 ± 2.7	13.9 ± 10.2	23/20–40	ELISA	Plasma	North
Gomez-Ambrosi et al., 2014	Spain	23.3 ± 1.6	4.4 ± 2.5	78/45.2 ± 13.8	39.4 ± 9.2	30.1 ± 21.4	84/44.4 ± 9.9	RIA	ND	South
Ekmen et al., 2016	Turkey	24 ± 1.3	2.53 ± 1.03	24/55.8 ± 8.8	31.5 ± 2.1	1.23 ± 0.73	23/48.5 ± 11.4	ELISA	Plasma	South
Maciver et al., 2015	USA	24.9 ± 2.9	4.6 ± 3.5	2309/47.7 ± 18.8	33.9 ± 4	11.5 ± 6.5	593/48.5 ± 16.2	RIA	Serum	South
Benbaibeche et al., 2014	Algeria	22 ± 2	3.25 ± 0.43	30/38 ± 3	32 ± 1	3.77 ± 2.05	30/44 ± 3	ELISA	ND	South
Esteghamati et al., 2011	Iran	23.65 ± 3.8	3.41 ± 2.37	907/41.53 ± 12.02	28.73 ± 4.07	5.1 ± 3.42	561/47.19 ± 1.95	ELISA	Serum	South
Soderberg et al., 2007	Australia	23 ± 0.2	3.8 ± 0.4	821/40.3	25.5 ± 0.36	5.6 ± 0.2	211/42.8	RIA	Serum	South
Katsuki et al., 2001	Japan	22.2 ± 0.5	3.6 ± 0.3	15/40.6 ± 1.7	29.2 ± 0.6	7.4 ± 0.8	15/38.2 ± 0.9	RIA	Serum	South
Haffner et al., 1997	USA	24.4 ± 0.3	3.3 ± 0.4	56/54.3 ± 0.7	29.7 ± 0.3	4.9 ± 0.5	31/53.9 ± 0.9	RIA	Serum	South
Vega et al., 2013	USA	24.1 ± 2.3	2.3 ± 2.2	55/53.0 ± 8.9	27.9 ± 2.5	10.4 ± 7.5	226/54.1 ± 10.2	RIA	Plasma	South

Note: ND—no data, USA—United State of America, RSA—Republic of South Africa.
